# The Relationship Between Nurses’ Perceptions of Disaster Preparedness and Psychological Preparedness: A Cross-Sectional Study

**DOI:** 10.3390/bs16081435

**Published:** 2026-08-20

**Authors:** Mehtap Genc, Ozge Cetinkaya, Emre Ciydem

**Affiliations:** 1 Department of Mental Health and Psychiatric Nursing, Faculty of Health Sciences, Karamanoğlu Mehmetbey University, 70100 Karaman, Türkiye; mehtap_coplu@kmu.edu.tr; 2 Balıkesir State Hospital, 10020 Balıkesir, Türkiye; ozgecetinkaya82@gmail.com; 3 Department of Mental Health and Psychiatric Nursing, Faculty of Nursing, Istanbul University, 34452 İstanbul, Türkiye; 4 The Faculty of Health Sciences, Bandırma Onyedi Eylül University, 10250 Bandırma, Türkiye

**Keywords:** disaster, natural disasters, disaster planning, relief work, disater nursing

## Abstract

Nurses’ psychological preparedness is an important component of effective disaster response; however, its relationship with preparedness perceptions across disaster management phases remains insufficiently understood. This cross-sectional correlational study examined the relationship between disaster preparedness perceptions and psychological preparedness among 237 nurses working in three hospitals in Balıkesir, Türkiye. Participants were selected using purposive sampling. Data were collected using the Disaster Preparedness Perception Scale and the Psychological Preparedness for Disaster Threat Scale. Descriptive statistics, Pearson correlation, and hierarchical multiple regression analyses were performed. The mean psychological preparedness score was 63.16 ± 11.88. Psychological preparedness was positively correlated with overall disaster preparedness perception (r = 0.697, *p* < 0.001), response-phase preparedness (r = 0.735, *p* < 0.001), and post-disaster preparedness (r = 0.650, *p* < 0.001), but not preparedness-phase perception (r = 0.080, *p* = 0.220). In the final regression model, response-phase (β = 0.480, *p* < 0.001) and post-disaster preparedness perceptions (β = 0.321, *p* < 0.001) were positively associated with psychological preparedness. Nurses without disaster training had lower psychological preparedness (β = −0.107, *p* = 0.017), as did nurses with postgraduate education compared with those with high school education (β = −0.167, *p* = 0.037). The final model explained 60.2% of the variance (adjusted R^2^ = 0.602). These findings highlight the relevance of disaster preparedness perceptions and training to nurses’ psychological preparedness and may inform institutional training and support strategies.

## 1. Introduction

Disasters are events that disrupt the normal functioning of societies, adversely affect human life, health, and socioeconomic structures, and exceed the capacity of available resources (Altuntaş et al., 2023; Sohn, 2015). In recent years, the increasing frequency and severity of natural and human-induced disasters worldwide have made the importance of disaster preparedness more evident (Luo et al., 2025). Disasters such as earthquakes, floods, wildfires, landslides, and industrial accidents place a substantial burden on healthcare systems and necessitate that healthcare professionals be adequately prepared for disaster situations (Feng et al., 2025; Ulupınar et al., 2024). In Türkiye, which is highly vulnerable to various types of disasters due to its geographical location, recent disaster events have clearly demonstrated the critical role of healthcare professionals in disaster management (Ulupınar et al., 2024).

Nurses constitute the largest professional group within healthcare systems and assume critical responsibilities during the preparedness, response, and recovery phases of disaster management (Luo et al., 2025). In disaster situations, nurses are expected not only to provide clinical care but also to perform triage, coordinate healthcare services, and address the physical and psychosocial needs of individuals and communities (Altuntaş et al., 2026; Said et al., 2022). Therefore, ensuring adequate disaster preparedness among nurses is essential for maintaining effective and uninterrupted healthcare services during disaster situations (Said & Chiang, 2020).

Balıkesir, where this study was conducted, is exposed to multiple natural and technological disaster risks due to its geographical, geological, and industrial characteristics. Its location within the influence of major fault systems creates a significant earthquake risk (Gündoğdu et al., 2020), while floods, storms, wildfires, and coastal hazards also affect the region (Erdem, 2013; Gençay & Karadere, 2024). In addition, its industrial infrastructure and related activities create potential technological hazards, including industrial accidents, fires, and hazardous material incidents (Özbey & Bayraktar, 2023; Silei, 2014). Therefore, Balıkesir’s multi-hazard context provides an important setting for assessing disaster preparedness and psychological preparedness among nurses.

Disaster preparedness is a multidimensional concept encompassing knowledge, skills, attitudes, and behaviors (Özcan, 2013). Although numerous studies have examined nurses’ disaster management competencies, disaster preparedness, and disaster response skills, disaster preparedness is not limited to technical knowledge and practical competencies alone (Feng et al., 2025; Ulupınar et al., 2024). During disasters, healthcare professionals face numerous psychological challenges, including heavy workloads, uncertainty, exposure to death and injuries, resource shortages, and high levels of stress. Therefore, psychological preparedness for disasters is recognized as a critical component of disaster management (Said & Chiang, 2020).

Psychological preparedness is defined as an individual’s ability to anticipate, manage, and adapt to the emotional, cognitive, and behavioral responses that may arise before, during, and after a disaster (Türkdoğan Görgün et al., 2023). Individuals who are psychologically prepared are reported to cope more effectively with disaster-related stress, maintain their functioning during crises, and adapt more rapidly in the aftermath of disasters (Duan et al., 2022; Huang et al., 2022). Indeed, a systematic review conducted by Said and Chiang (2020) reported that nurses’ disaster preparedness generally ranged from low to moderate levels and emphasized that strengthening psychological preparedness is of critical importance in disaster management. In addition, individual psychological resources such as self-efficacy, resilience, hope, optimism, and coping skills have been identified as important determinants of psychological preparedness (Özdemir et al., 2025; Şermet Kaya & Erdoğan, 2025; Duan et al., 2022). From a nursing perspective, psychological preparedness is important not only for managing nurses’ own emotional responses but also for enabling them to provide psychosocial support to individuals affected by disasters (Said & Chiang, 2020). One of the factors influencing psychological preparedness among nurses is disaster preparedness perception (Altuntaş et al., 2026).

Disaster preparedness perception refers to how individuals evaluate their knowledge, skills, and competencies related to disaster situations (Özcan, 2013). Individuals with higher levels of disaster preparedness perception are expected to demonstrate more effective behaviors during disasters and manage crisis situations more successfully (Feng et al., 2025; Ulupınar et al., 2024). To the best of our knowledge, no study has directly examined the relationship between the subdimensions of disaster preparedness perception and psychological preparedness among nurses. Nevertheless, studies have investigated the relationships between disaster preparedness, disaster response self-efficacy, psychological capital, and psychological preparedness. Previous research has reported that disaster response self-efficacy and psychological capital are positively associated with psychological preparedness, whereas disaster risk perception may not be significantly related to psychological preparedness (Duan et al., 2022; Özdemir et al., 2025; Altuntaş et al., 2026).

Psychological preparedness for disasters plays a crucial role in enabling individuals to cope effectively, adapt, and maintain functioning during disaster situations. However, findings regarding the factors influencing psychological preparedness remain limited and inconsistent. Considering the critical roles of nurses throughout disaster processes, identifying the individual and professional factors associated with psychological preparedness is particularly important (Said & Chiang, 2020; Alrabie et al., 2025; Koyama et al., 2024; Nikitara et al., 2025). Determining these factors may contribute to the development of disaster education programs, the identification of at-risk groups, and the enhancement of nurses’ psychological preparedness for disasters. Therefore, this study aimed to examine the relationship between nurses’ disaster preparedness perceptions and their psychological preparedness for disaster threats. Specifically, the study sought to determine whether nurses’ perceptions of preparedness during the preparedness, response, and post-disaster phases were associated with psychological preparedness and to identify the individual and professional factors associated with psychological preparedness. Accordingly, the following research questions were addressed: (1) Are nurses’ disaster preparedness perceptions and their subdimensions associated with psychological preparedness for disaster threats? (2) Which individual, professional, and disaster preparedness-related factors are associated with nurses’ psychological preparedness for disaster threats?

## 2. Materials and Methods

### 2.1. Design

This study was conducted using a correlational design. The Strengthening the Reporting of Observational Studies in Epidemiology (STROBE) guideline was used to enhance the reporting quality of this observational study (von Elm et al., 2007; Babaoğlu et al., 2021).

### 2.2. Population and Sample

The study was conducted in Balıkesir, a province exposed to multiple natural and technological disaster risks, including earthquakes, floods, wildfires, and industrial hazards. The study population consisted of nurses working in three hospitals located in Balıkesir, Türkiye, between June and December 2025. These hospitals included one public hospital, one university hospital, and one private hospital. The minimum sample size required to represent the target population was determined using G*Power version 3.1.9.2 (Heinrich Heine University Düsseldorf, Düsseldorf, Germany) (Faul et al., 2007). Since no previous study examining similar variables was identified in the literature, a pilot study involving 35 participants was conducted to estimate the required sample size. Based on the results of the multiple linear regression analysis performed in the pilot study, the effect size (f^2^) was calculated using the explanatory power of the model (R^2^) and was determined to be 0.20. For the sample size calculation, 16 predictor parameters were specified, corresponding to the exact parameterization planned for the final multiple linear regression model. This count included continuous predictors and individual dummy-coded parameters for categorical variables, with one category designated as the reference for each categorical predictor. Specifically, education level contributed three dummy variables, department contributed two, and institution type contributed two, while binary variables each contributed one parameter. Thus, the G*Power calculation was based on the same 16 predictor parameters represented in the final comprehensive regression model. Accordingly, assuming a 95% confidence level (1 − α), 95% statistical power (1 − β), an effect size (f^2^) of 0.20, and 16 predictors, the minimum required sample size was calculated as 156 participants. However, to enable a more comprehensive examination of the relationships among the study variables, purposive sampling was used for participant selection. A total of 248 nurses completed the data collection forms. Four attention-check items were embedded in the questionnaire to assess response attentiveness. Participants were instructed to select a specific response option for each item (i.e., “Strongly agree,” “Agree,” “Disagree,” and “Neither agree nor disagree,” respectively). The exclusion criterion was predefined, and participants who responded incorrectly to at least one of the four attention-check items were excluded from the analyses. Accordingly, 11 participants were excluded, resulting in a final analytical sample of 237 nurses. The exact number of nurses approached across the three hospitals was not recorded; therefore, a formal response rate could not be calculated.

### 2.3. Inclusion and Exclusion Criteria

The inclusion criteria were being willing to participate in the study, being between 18 and 65 years of age, having worked as a nurse for at least six months, having direct patient care as the primary professional responsibility, being actively employed in one of the hospitals where the study was conducted, and not being on leave or medical leave during the data collection period.

The exclusion criteria included having less than six months of nursing experience, holding an administrative position without direct patient care responsibilities, providing incomplete data in the study questionnaires, and being on leave, medical leave, or otherwise absent from duty during the data collection period.

### 2.4. Instruments

Information Form: The form developed by the researchers based on the relevant literature consists of eight questions assessing nurses’ sociodemographic characteristics and disaster exposure experiences (Altuntaş et al., 2026; Pires et al., 2025; Hong et al., 2025; Chegini et al., 2022).

Psychological Preparedness for Disaster Threat Scale: The Psychological Preparedness for Disaster Threat Scale was developed by Zulch (2019) to assess individuals’ psychological preparedness for disasters and was adapted into Turkish by Türkdoğan Görgün et al. (2023). The scale consists of 21 items and three subdimensions: knowledge and management of the external situational environment (Items 1–9), management of emotional and psychological responses (Items 10–16, 20, and 21), and management of the social environment (Items 17–19). All items are rated on a 4-point Likert scale ranging from 1 (strongly disagree) to 4 (strongly agree). Each item is scored between 1 and 4, and there are no reverse-scored items. Subscale scores are calculated by summing the items belonging to each subdimension separately. In addition, a total scale score can be obtained by combining the subscale scores. The possible score ranges are 9–36 for the knowledge and management of the external situational environment subscale, 9–36 for the management of emotional and psychological responses subscale, and 3–12 for the management of the social environment subscale. The scale does not have a predetermined cut-off score, and scores are not directly classified as high, moderate, or low psychological preparedness. However, higher scores indicate greater psychological preparedness for disaster threats. In the Turkish adaptation study, Cronbach’s alpha coefficients for the subscales ranged from 0.83 to 0.93 (Türkdoğan Görgün et al., 2023). In the present study, Cronbach’s alpha coefficients for the subscales ranged from 0.86 to 0.94, while Cronbach’s alpha for the total Psychological Preparedness for Disaster Threat Scale was 0.92.

Disaster Preparedness Perception Scale for Nurses: The Disaster Preparedness Perception Scale for Nurses was developed in Turkish by Özcan (2013) to assess nurses’ perceptions of disaster preparedness. The scale consists of 20 items and three subdimensions: preparedness phase (Items 1–6), response phase (Items 7–15), and post-disaster phase (Items 16–20). The scale uses a 5-point Likert response format ranging from 1 (strongly disagree) to 5 (strongly agree) and is completed through self-report. There are no reverse-scored items and no established cut-off score. Higher scores indicate higher levels of disaster preparedness perception among nurses. Both subscale scores and a total scale score can be calculated. In the original scale development study, Cronbach’s alpha coefficients for the subscales ranged from 0.87 to 0.89, while the overall Cronbach’s alpha coefficient was reported as 0.91 (Özcan, 2013). In the present study, Cronbach’s alpha coefficients ranged from 0.85 to 0.93 for the subscales, and the overall Cronbach’s alpha coefficient was 0.92.

### 2.5. Procedure

Data were collected by the researcher through face-to-face interviews between June and December 2025, following approval from the ethics committee and the acquisition of all necessary institutional permissions. Prior to data collection, participants were provided with detailed information regarding the purpose and scope of the study, and informed consent was obtained after emphasizing that participation was entirely voluntary. Throughout the study, careful attention was given to protecting participants’ privacy and confidentiality, and all data were recorded in accordance with confidentiality principles. The administration of the data collection instruments took approximately 20–30 min per participant.

### 2.6. Analysis

The data obtained from the study were analyzed using IBM SPSS Statistics for Windows, version 23.0 (IBM Corp., Armonk, NY, USA).The distribution of continuous variables was assessed using the Kolmogorov–Smirnov test, skewness and kurtosis values, and visual inspection. Descriptive statistics were presented using frequencies, percentages, means, standard deviations, and minimum and maximum values. The distribution of continuous variables was assessed using the Kolmogorov–Smirnov test and skewness and kurtosis values. The Kolmogorov–Smirnov test indicated significant deviations from normality for psychological preparedness (D = 0.102, *p* < 0.001), preparedness-phase perception (D = 0.190, *p* < 0.001), response-phase perception (D = 0.078, *p* = 0.001), post-disaster-phase perception (D = 0.102, *p* < 0.001), and total disaster preparedness perception (D = 0.071, *p* = 0.006). Skewness values ranged from −2.109 to −0.117 and kurtosis values from −0.006 to 8.136. Pearson correlation analysis was conducted to examine the relationships between disaster preparedness perception and psychological preparedness for disaster threats among nurses. Given the observed deviations from normality, Spearman’s rank correlation analysis was additionally performed as a sensitivity analysis to assess the robustness of the Pearson correlation findings. Sensitivity analysis using Spearman’s rank correlations yielded the same pattern of statistical significance as the Pearson correlation analysis. In addition, hierarchical multiple linear regression analysis was performed to identify factors associated with psychological preparedness for disaster threats. Variables were entered sequentially in predefined blocks, and changes in R^2^ and adjusted R^2^ were evaluated across models. Statistical significance was set at *p* < 0.05. Regression assumptions were assessed before interpreting the models. Multicollinearity was evaluated using correlations, tolerance, and variance inflation factor (VIF) values. Because age and years of nursing experience were highly correlated (r = 0.925, *p* < 0.001), years of nursing experience was excluded from the regression models. In the final model, tolerance values ranged from 0.190 to 0.933 and VIF values from 1.072 to 5.260, indicating no evidence of severe multicollinearity. The Durbin–Watson statistic was 1.912. Visual inspection of the residual histogram, normal P–P plot, and standardized residual-versus-predicted-value plot indicated approximate residual normality, linearity, and homoscedasticity. The maximum Cook’s distance was 0.108, indicating no highly influential observations.

### 2.7. Ethical Issues

This study was conducted in accordance with scientific ethical principles and relevant legal regulations. Prior to the initiation of the study, ethical approval was obtained from the Non-Interventional Research Ethics Committee of Bandırma Onyedi Eylül University Health Sciences Faculty. The study, registered under application number 2025-46, was approved during the committee meeting numbered 2025-03 held on 10 March 2025. Following ethical approval, all necessary institutional permissions were obtained from the participating institutions. Permission to collect data in the public hospital was granted by the Provincial Health Directorate following the unanimous decision of the Scientific Research Requests Commission on 22 May 2025. Permission to conduct the survey in the university hospital was obtained through official document number E-26489196-900-512730 dated 5 May 2025. Institutional approval from the private hospital was obtained on 28 April 2025. Copyright and usage rights related to all measurement instruments used in the study were respected, and written permission was obtained from the scale developers via email correspondence. All nurses who agreed to participate were thoroughly informed about the purpose, scope, procedures, and potential implications of the study. Written and verbal informed consent was obtained from each participant through the Informed Consent Form. The confidentiality of participants’ personal data was strictly protected throughout the study. Respect for human dignity and the principle of voluntary participation were maintained at all stages of the research. The study was conducted in accordance with the principles of the Declaration of Helsinki.

## 3. Results

The distribution of the participants’ individual and disaster-related characteristics is presented in Table 1. The mean age of the nurses was 35.74 ± 9.92 years (min: 18, max: 60). Most participants were female (82.7%), held a bachelor’s degree (71.7%), and were married (60.8%). In addition, 61.6% were working in emergency departments or intensive care units (Table 1). It was found that 42.2% of the nurses had previously experienced a disaster, with earthquakes being the most commonly reported disaster type. Among the participants, 79.7% had received disaster management training; of those, 55.7% had received theoretical training only, while 24.1% had received both theoretical and practical training. The mean duration of professional experience was 13.71 ± 10.37 years (min: 1, max: 31), and 73.8% of the participants were employed in public hospitals (Table 1).

The mean scores of the nurses on the Psychological Preparedness for Disaster Threat Scale and the Disaster Preparedness Perception Scale for Nurses are presented in Table 2. The mean total score on the Psychological Preparedness for Disaster Threat Scale was 63.15 ± 11.87. The mean total score on the Disaster Preparedness Perception Scale for Nurses was 3.76 ± 0.53. Regarding the subdimensions of the Disaster Preparedness Perception Scale, the mean score for the preparedness phase perception subscale was 4.42 ± 0.65, the mean score for the response phase perception subscale was 3.41 ± 0.70, and the mean score for the post-disaster phase perception subscale was 3.64 ± 0.76 (Table 2).

The relationships between disaster preparedness perception and psychological preparedness for disaster threats among nurses are presented in Table 3. A strong positive correlation was found between the total score of the Disaster Preparedness Perception Scale for Nurses and psychological preparedness (r = 0.697, r^2^ = 0.486, *p* < 0.001), indicating 48.6% shared variance. In addition, preparedness perception related to the response phase (r = 0.735, r^2^ = 0.540, *p* < 0.001) and preparedness perception related to the post-disaster phase (r = 0.650, r^2^ = 0.423, *p* < 0.001) were both strongly and positively associated with psychological preparedness, corresponding to 54.0% and 42.3% shared variance, respectively. In contrast, no statistically significant relationship was found between preparedness perception related to the preparedness phase and psychological preparedness (r = 0.080, r^2^ = 0.006, *p* = 0.220) (Table 3).

The results of the hierarchical regression analysis conducted to identify factors associated with psychological preparedness for disaster threats among nurses are presented in Table 4. In Model 1, individual and professional variables were included. The model was statistically significant (F(12, 224) = 3.433, *p* < 0.001) and explained 11.0% of the variance in psychological preparedness (Adj. R^2^ = 0.110). In Model 2, preparedness perception related to the post-disaster phase was added, resulting in a substantial increase in the explanatory power of the model (F(13, 223) = 18.691, *p* < 0.001; Adj. R^2^ = 0.494). In Model 3, preparedness perception related to the response phase was added, further increasing the explanatory power of the model (F(14, 222) = 26.536, *p* < 0.001; Adj. R^2^ = 0.602). Finally, preparedness perception related to the preparedness phase was added in Model 4; however, this variable did not make a significant contribution to the model (B = −0.818, SE = 0.799, β = −0.045, t = −1.024, *p* = 0.307, 95% CI [−2.393, 0.757]).

The final model explained 60.2% of the variance in psychological preparedness for disaster threats (F(15, 221) = 24.842, *p* < 0.001; Adj. R^2^ = 0.602). Preparedness perception related to the post-disaster phase was positively associated with psychological preparedness (B = 5.002, SE = 0.926, β = 0.321, t = 5.402, *p* < 0.001, 95% CI [3.177, 6.828]). Similarly, preparedness perception related to the response phase was positively associated with psychological preparedness (B = 8.052, SE = 1.023, β = 0.480, t = 7.873, *p* < 0.001, 95% CI [6.036, 10.067]). Nurses who had not received disaster training had psychological preparedness scores 3.167 points lower than those who had received disaster training (B = −3.167, SE = 1.316, β = −0.107, t = −2.406, *p* = 0.017, 95% CI [−5.761, −0.573]). In addition, postgraduate education was negatively associated with psychological preparedness compared with high school education (B = −5.799, SE = 2.767, β = −0.167, t = −2.096, *p* = 0.037, 95% CI [−11.251, −0.347]). The remaining variables were not significantly associated with psychological preparedness (*p* > 0.05) (Table 4).

## 4. Discussion

In this study, preparedness perceptions related to the post-disaster and response phases were significantly associated with psychological preparedness among nurses. These findings suggest that perceiving oneself as competent to perform professional duties effectively during and after disasters is associated with higher levels of psychological preparedness for disaster situations. In contrast, no significant relationship was found between preparedness perception related to the preparedness phase and psychological preparedness. This finding suggests that knowledge and awareness regarding pre-disaster planning, training, and organizational processes alone may not necessarily be associated with higher levels of psychological preparedness.

No study was identified in the literature that directly examined the relationship between the subdimensions of disaster preparedness perception and psychological preparedness among nurses. Nevertheless, the present findings are partially consistent with studies investigating the relationships among disaster preparedness, self-efficacy, and psychological resources. In a study conducted with nursing students in Türkiye, Altuntaş et al. (2026) found no significant relationship between disaster risk perception and psychological preparedness. This finding supports the present study’s result regarding the preparedness phase, suggesting that perceiving disaster risk alone may not be sufficient for individuals to feel psychologically prepared.

On the other hand, a study conducted by Duan et al. (2022) among nurses working in public hospitals in China reported a significant positive relationship between disaster preparedness and psychological capital. Psychological capital encompasses positive psychological resources such as self-efficacy, hope, optimism, and resilience, which facilitate adaptation to challenging circumstances (Luthans & Youssef, 2004; Karatepe & Avci, 2017). From this perspective, the finding that preparedness perceptions related to the response and post-disaster phases increase psychological preparedness may be explained by the tendency of individuals with stronger psychological resources to perceive themselves as more capable and prepared in disaster situations.

Similarly, Özdemir et al. (2025) reported a significant positive relationship between disaster response self-efficacy and psychological preparedness for disaster threats among nursing students. This finding is consistent with the present study, in which preparedness perception related to the response phase emerged as an important predictor of psychological preparedness. Individuals who believe they can provide effective care and manage crises during disasters may also feel more psychologically prepared to cope with such situations.

Furthermore, in a study conducted with nurses, Şermet Kaya and Erdoğan (2025) found a positive relationship between nurses’ perceived competence regarding disaster-related duties and responsibilities and their disaster preparedness levels, whereas beliefs about disaster preparedness were not associated with competence. This finding is consistent with the present study, in which preparedness perception related to the preparedness phase was not associated with psychological preparedness, whereas perceptions related to the response and post-disaster phases were significant predictors. Therefore, enhancing nurses’ psychological preparedness may require not only increasing awareness and beliefs about disasters but also strengthening self-efficacy and practical competencies related to disaster response and recovery processes.

These findings suggest that psychological preparedness among nurses may be more closely associated with perceived competence in performing duties during and after disasters than with knowledge of pre-disaster planning and organizational processes. In other words, nurses’ psychological readiness may be more closely related to their perceived ability to provide care under crisis conditions, make rapid decisions, communicate effectively, and manage post-disaster recovery processes than to theoretical preparedness alone. This interpretation is consistent with the observed associations between psychological preparedness and preparedness perceptions related to the response and post-disaster phases, whereas no significant association was observed for preparedness perception related to the preparedness phase.

The present study also found that nurses who had not received disaster training demonstrated lower psychological preparedness than those who had received such training. This finding indicates an association between disaster training status and psychological preparedness among nurses, although the cross-sectional design does not allow conclusions regarding the direction or causality of this relationship. Consistent with the present results, numerous studies have reported that disaster training enhances disaster preparedness among healthcare professionals (Şermet Kaya & Erdoğan, 2025; Wang et al., 2023; Ediz & Yanik, 2024; Said et al., 2020). Increased knowledge and skills related to disaster management, crisis response, and post-disaster care gained through disaster training may help nurses feel more confident and psychologically prepared when confronted with disaster situations. However, some studies have reported no significant relationship between disaster training and psychological preparedness for disasters (Özdemir et al., 2025). Such inconsistencies may be attributable to differences in sample characteristics, the content and duration of training programs, levels of disaster experience, or the measurement instruments used.

An additional finding was that nurses with postgraduate education had lower psychological preparedness scores than those with a high school education after adjustment for other variables. This finding should be interpreted cautiously, particularly because the number of nurses in the reference education category was relatively small and the association was modest. Findings in the literature regarding education and disaster preparedness are not consistent. Wang et al. (2023) reported higher disaster preparedness among nurses with junior college and undergraduate education than among those with postgraduate education, which is broadly consistent with the direction of our finding. In contrast, Ediz and Yanik (2024) found no significant differences in disaster preparedness perceptions according to educational level, while Şermet Kaya and Erdoğan (2025) reported no significant association between educational level and General Disaster Preparedness Belief Scale scores. These differences may reflect variations in study populations, educational categories, measurement instruments, and the dimensions of disaster preparedness assessed. Importantly, higher academic education does not necessarily indicate greater disaster-specific training, practical disaster experience, or psychological readiness to respond to disaster threats. The lower psychological preparedness observed among postgraduate nurses in the present study may also reflect differences in professional roles, responsibilities, or awareness of disaster-related risks and complexities. However, given the cross-sectional design, the relatively small reference group, and the modest association observed, these explanations remain tentative. Further studies with more balanced educational groups are needed to clarify the relationship between educational attainment and psychological preparedness for disasters.

This study contributes to the literature by examining the relationship between psychological preparedness for disaster threats and disaster preparedness perception among nurses. The study’s strengths include its multicenter design involving nurses working in different healthcare institutions and its focus on a sample from a region exposed to multiple disaster risks. Furthermore, the use of hierarchical regression analysis enabled a comprehensive evaluation of the relationships among the study variables, thereby strengthening the scientific value of the study. Several limitations should be acknowledged. First, the cross-sectional design precludes establishing temporal or causal relationships between disaster preparedness perceptions and psychological preparedness. Second, purposive sampling may have introduced selection bias, and the 237 participants may not be fully representative of the broader nursing population; therefore, the generalizability of the findings should be interpreted with caution. Third, although nurses from three different types of hospitals were included, all participating institutions were located in a single province, which further limits the generalizability of the findings to nurses working in other geographical and healthcare settings. Fourth, the use of self-report measures may have introduced response and social desirability biases. Fifth, the exclusion of participants based on predefined attention-check criteria, although intended to improve data quality, may have introduced additional selection effects. Moreover, because the exact number of nurses approached was not recorded, the response rate could not be calculated, limiting the assessment of potential nonresponse bias. Finally, because psychological preparedness and disaster preparedness perceptions were assessed concurrently, the direction of the observed associations cannot be determined. Additionally, the relatively small number of nurses in some educational categories, particularly the high school reference group, may have reduced the precision of comparisons across educational levels; therefore, the observed association between postgraduate education and psychological preparedness should be interpreted cautiously.

## 5. Conclusions

This study found that psychological preparedness for disaster threats among nurses was significantly associated with preparedness perceptions related to the response and post-disaster phases. In addition, nurses who had not received disaster training demonstrated lower levels of psychological preparedness than those who had received disaster training, while nurses with postgraduate education had lower psychological preparedness scores than those with a high school education. Given the cross-sectional design of the study, these findings indicate associations and do not establish causal relationships. The observed associations between disaster training status, preparedness perceptions, and psychological preparedness may inform the development of institutional disaster preparedness programs. Such programs may consider incorporating content related to psychosocial support, stress management, and crisis management within the scope of psychiatric and mental health nursing. At the institutional level, hospital and nurse managers may also consider periodically assessing nurses’ psychological preparedness to identify additional training and support needs. At the policy level, disaster preparedness policies may consider incorporating psychological preparedness and psychosocial support competencies into disaster-related training frameworks for nurses. Future longitudinal studies are recommended to examine changes in psychological preparedness over time, clarify the temporal relationships between disaster training, preparedness perceptions, and psychological preparedness, and further investigate the role of educational level using larger and more balanced educational groups.

## Figures and Tables

**Table 1:** Distribution of nurses’ individual and disaster-related characteristics (n = 237).

Variables	Number (n)	Percentage (%)
**Gender**		
Female	196	82.7
Male	41	17.3
**Age** (Mean: 35.74 ± 9.92, Min–Max: 18–60)		
**Working duration (years)** (Mean: 13.71 ± 10.37, Min–Max: 1–31)		
**Education level**		
High school	12	5.1
Associate degree	23	9.7
Bachelor’s degree	170	71.7
Postgraduate	32	13.5
**Marital status**		
Married	144	60.8
Single/Divorced/Widowed	93	39.2
**Department worked in**		
Emergency and intensive care	146	61.6
Inpatient treatment services	67	28.3
Outpatient diagnosis and treatment units	24	10.1
**Disaster exposure status**		
Yes	100	42.2
No	137	57.8
**Type**		
Avalanche	3	3
Earthquake	97	97
Mine explosion	2	2
Flood	3	3
Fire	4	4
War	1	1
**Disaster management training status**		
Yes	189	79.7
No	48	20.3
**Type of training**		
Theoretical	132	55.7
Theoretical and Practical	57	24.1
**Type of hospital**		
State hospital	175	73.8
University hospital	41	17.3
Private hospital	21	8.9

**Table 2:** Nurses’ mean scores on the scales.

Scales	Mean	±SD	Min.	Max.
Psychological preparedness for disaster threat scale total score	63.15	11.87	21	84
Nurses’ disaster preparedness perception scale total score	3.76	0.53	1.20	4.90
Preparedness perception regarding the preparedness phase	4.42	0.65	1	5
Preparedness perception regarding the response phase	3.41	0.70	1.30	5
Preparedness perception regarding the post-disaster phase	3.64	0.76	1	5

**Table 3:** Relationships between nurses’ disaster preparedness perception and their level of psychological preparedness for disaster threat (n = 237).

Variables		Psychological Preparedness for Disaster Threat Scale Total Score
Nurses’ disaster preparedness perception scale total score	r	0.697
	*p*	<0.001
	r^2^	0.486
Preparedness perception regarding the preparedness phase	r	0.080
	*p*	0.220
	r^2^	0.006
Preparedness perception regarding the response phase	r	0.735
	*p*	<0.001
	r^2^	0.540
Preparedness perception regarding the post-disaster phase	r	0.650
	*p*	<0.001
	r^2^	0.423

Note: r: Pearson correlation coefficient.

**Table 4:** Factors affecting nurses’ level of psychological preparedness for disaster threat (n = 237).

**Model 1 (F(12, 224) = 3.433, *p* < 0.001; Adj. R^2^ = 0.110)**									
**Variables**	**Unstandardized Coefficient**		**Standardized Coefficient**	**t**	***p***	**95% CI**		**Collinearity Statistics**	
	**B**	**SE**	**Beta**			**Lower**	**Upper**	**Tolerence**	**VIF**
(Constant)	73.180	5.085		14.391	0.000	63.160	83.201		
Age (years)	−0.044	0.090	−0.037	−0.488	0.626	−0.221	0.133	0.668	1.498
Gender = Male	4.275	1.992	0.136	2.146	0.033	0.349	8.201	0.933	1.072
Marital status = Single/Divorced/Widowed	−1.638	1.694	−0.067	−0.967	0.335	−4.976	1.700	0.774	1.291
Education level = Associate degree	−4.521	4.154	−0.113	−1.088	0.278	−12.707	3.665	0.350	2.854
Education level = Bachelor’s degree	−6.399	3.665	−0.243	−1.746	0.082	−13.622	0.823	0.195	5.141
Education level = Postgraduate	−6.430	4.113	−0.185	−1.563	0.119	−14.537	1.676	0.268	3.730
Department worked = Inpatient treatment services	−2.450	1.784	−0.093	−1.373	0.171	−5.967	1.066	0.821	1.219
Department worked = Outpatient diagnosis and treatment units	−0.355	2.494	−0.009	−0.142	0.887	−5.269	4.559	0.936	1.068
Disaster exposure status = No	−1.915	1.576	−0.080	−1.215	0.225	−5.020	1.190	0.875	1.143
Disaster training status = No	−8.021	1.918	−0.272	−4.183	0.000	−11.799	−4.242	0.892	1.121
Institution type worked = University hospital	1.229	2.120	0.039	0.580	0.563	−2.948	5.406	0.824	1.213
Institution type worked = Private hospital	6.674	2.959	0.160	2.255	0.025	0.842	12.506	0.749	1.335
**Model 2—Post-Disaster Phase (F(13, 223) = 18.691, *p* < 0.001; Adj. R^2^ = 0.494)**									
**Variables**	**Unstandardized Coefficient**		**Standardized Coefficient**	**t**	***p***	**95% CI**		**Collinearity Statistics**	
	**B**	**SE**	**Beta**			**Lower**	**Upper**	**Tolerence**	**VIF**
(Constant)	34.554	4.844		7.134	0.000	25.009	44.099		
Age (years)	0.007	0.068	0.006	0.110	0.913	−0.126	0.141	0.666	1.503
Gender = Male	4.263	1.503	0.136	2.836	0.005	1.301	7.225	0.933	1.072
Marital status = Single/Divorced/Widowed	−1.891	1.278	−0.078	−1.480	0.140	−4.410	0.628	0.774	1.292
Education level = Associate degree	−1.709	3.141	−0.043	−0.544	0.587	−7.899	4.481	0.349	2.867
Education level = Bachelor’s degree	−6.221	2.765	−0.236	−2.250	0.025	−11.670	−0.773	0.195	5.141
Education level = Postgraduate	−8.064	3.106	−0.232	−2.596	0.010	−14.184	−1.943	0.268	3.736
Department worked = Inpatient treatment services	−2.270	1.346	−0.086	−1.687	0.093	−4.923	0.382	0.821	1.219
Department worked = Outpatient diagnosis and treatment units	0.077	1.881	0.002	0.041	0.967	−3.631	3.784	0.936	1.068
Disaster exposure status = No	−1.263	1.190	−0.053	−1.062	0.289	−3.608	1.081	0.873	1.145
Disaster training status = No	−4.097	1.477	−0.139	−2.773	0.006	−7.008	−1.185	0.855	1.169
Institution type worked = University hospital	1.625	1.599	0.052	1.016	0.311	−1.527	4.777	0.824	1.214
Institution type worked = Private hospital	2.524	2.255	0.061	1.119	0.264	−1.920	6.968	0.734	1.362
Preparedness perception regarding the post-disaster phase	9.803	0.751	0.630	13.061	0.000	8.324	11.282	0.923	1.083
**Model 3—Post-Disaster Phase, Response Phase (F(14, 222) = 26.536, *p* < 0.001; Adj. R^2^ = 0.602)**									
**Variables**	**Unstandardized Coefficient**		**Standardized Coefficient**	**t**	***p***	**95% CI**		**Collinearity Statistics**	
	**B**	**SE**	**Beta**			**Lower**	**Upper**	**Tolerence**	**VIF**
(Constant)	21.008	4.624		4.543	0.000	11.895	30.120		
Age (years)	0.027	0.060	0.023	0.450	0.653	−0.092	0.146	0.664	1.505
Gender = Male	1.047	1.393	0.033	0.752	0.453	−1.698	3.792	0.853	1.173
Marital status = Single/Divorced/Widowed	−0.897	1.139	−0.037	−0.787	0.432	−3.143	1.348	0.765	1.308
Education level = Associate degree	−0.014	2.792	0.000	−0.005	0.996	−5.516	5.487	0.347	2.885
Education level = Bachelor’s degree	−3.288	2.478	−0.125	−1.327	0.186	−8.172	1.596	0.190	5.260
Education level = Postgraduate	−5.865	2.766	−0.169	−2.120	0.035	−11.316	−0.414	0.265	3.774
Department worked = Inpatient treatment services	−0.754	1.208	−0.029	−0.624	0.533	−3.136	1.627	0.800	1.250
Department worked = Outpatient diagnosis and treatment units	−0.161	1.667	−0.004	−0.096	0.923	−3.447	3.125	0.936	1.069
Disaster exposure status = No	−0.241	1.062	−0.010	−0.226	0.821	−2.334	1.853	0.860	1.162
Disaster training status = No	−3.257	1.314	−0.110	−2.479	0.014	−5.845	−0.668	0.850	1.177
Institution type worked = University hospital	0.800	1.421	0.026	0.563	0.574	−2.001	3.601	0.819	1.220
Institution type worked = Private hospital	2.092	1.999	0.050	1.047	0.296	−1.847	6.031	0.734	1.363
Preparedness perception regarding the post-disaster phase	4.855	0.915	0.312	5.306	0.000	3.052	6.658	0.488	2.051
Preparedness perception regarding the response phase	8.055	1.023	0.481	7.875	0.000	6.040	10.071	0.452	2.210
**Model 4—Post-Disaster Phase, Response Phase, Preparedness Phase (F(15, 221) = 24.842, *p* < 0.001; Adj. R^2^ = 0.602)**									
**Variables**	**Unstandardized Coefficient**		**Standardized Coefficient**	**t**	***p***	**95% CI**		**Collinearity Statistics**	
	**B**	**SE**	**Beta**			**Lower**	**Upper**	**Tolerence**	**VIF**
(Constant)	24.078	5.511		4.369	0.000	13.217	34.938		
Age (years)	0.025	0.060	0.021	0.416	0.678	−0.094	0.144	0.664	1.507
Gender = Male	1.029	1.393	0.033	0.739	0.461	−1.716	3.774	0.853	1.173
Marital status = Single/Divorced/Widowed	−0.893	1.139	−0.037	−0.784	0.434	−3.138	1.353	0.765	1.308
Education level = Associate degree	0.088	2.793	0.002	0.032	0.975	−5.416	5.593	0.346	2.888
Education level = Bachelor’s degree	−3.279	2.478	−0.125	−1.323	0.187	−8.162	1.604	0.190	5.260
Education level = Postgraduate	−5.799	2.767	−0.167	−2.096	0.037	−11.251	−0.347	0.265	3.776
Department worked = Inpatient treatment services	−0.552	1.224	−0.021	−0.451	0.653	−2.965	1.861	0.779	1.284
Department worked = Outpatient diagnosis and treatment units	−0.062	1.670	−0.002	−0.037	0.971	−3.353	3.230	0.933	1.072
Disaster exposure status = No	−0.315	1.065	−0.013	−0.296	0.767	−2.413	1.783	0.856	1.168
Disaster training status = No	−3.167	1.316	−0.107	−2.406	0.017	−5.761	−0.573	0.846	1.182
Institution type worked = University hospital	0.911	1.425	0.029	0.639	0.523	−1.897	3.720	0.815	1.228
Institution type worked = Private hospital	2.239	2.004	0.054	1.118	0.265	−1.710	6.189	0.730	1.370
Preparedness perception regarding the post-disaster phase	5.002	0.926	0.321	5.402	0.000	3.177	6.828	0.476	2.102
Preparedness perception regarding the response phase	8.052	1.023	0.480	7.873	0.000	6.036	10.067	0.452	2.210
Preparedness perception regarding the preparedness phase	−0.818	0.799	−0.045	−1.024	0.307	−2.393	0.757	0.879	1.138

Note: Variables included in the regression model: age, disaster training status (reference category: yes), gender (reference category: female), department worked—inpatient treatment services (reference category: emergency and intensive care), department worked—outpatient diagnosis and treatment units (reference category: emergency and intensive care), disaster exposure status (reference category: yes), education level—associate degree (reference category: high school), education level—bachelor’s degree (reference category: high school), education level—postgraduate (reference category: high school), marital status (reference category: married), institution type worked—university hospital (reference category: state hospital), institution type worked—private hospital (reference category: state hospital).

## Data Availability

The data presented in this study are available on request from the corresponding author. The data are not publicly available due to ethical and privacy restrictions.

## References

[B1-behavsci-16-01435] Alrabie T., Brown M., Rice B., Marsh L. (2025). Disaster preparedness and response among healthcare professionals during the hajj: A systematic literature review. Healthcare.

[B2-behavsci-16-01435] Altuntaş S., Aydin D., Kuleyin B., Sati Kirkan T. (2026). The impact of disaster risk perception on psychological preparedness and response self-efficacy in nursing students: A cross-sectional study. Psychology, Health & Medicine.

[B3-behavsci-16-01435] Altuntaş S., Korkmaz A. Ç., Efe N., Demirtaş H., Kuleyin B. (2023). Problems experienced by nurses working in the region affected by Kahramanmaraş earthquakes in Turkey and their recommendations for risk reduction: A descriptive and cross-sectional study. International Journal of Disaster Risk Reduction.

[B4-behavsci-16-01435] Babaoğlu A. B., Tekindal M., Büyükuysal M. Ç., Tözün M., Elmalı F., Bayraktaroğlu T., Tekindal M. A. (2021). Epidemiyolojide gözlemsel çalışmaların raporlanması: STROBE kriterlerinin Türkçe uyarlaması. Batı Karadeniz Tıp Dergisi.

[B5-behavsci-16-01435] Chegini Z., Arab-Zozani M., Kakemam E., Lotfi M., Nobakht A., Aziz Karkan H. (2022). Disaster preparedness and core competencies among emergency nurses: A cross-sectional study. Nursing Open.

[B6-behavsci-16-01435] Duan Y., He J., Zheng R., Feng X., Xiao H. (2022). The relationship between disaster preparedness, psychological capital, and coping style among nurses: A cross-sectional study from China. Perspectives in Psychiatric Care.

[B7-behavsci-16-01435] Ediz Ç., Yanik D. (2024). Disaster preparedness perception, pyschological resiliences and empathy levels of nurses after 2023 Great Turkiye earthquake: Are nurses prepared for disasters: A risk management study. Public Health Nursing.

[B8-behavsci-16-01435] Erdem U. (2013). Yerleşimlerin taşıdığı deniz taşkını, sel ve deprem afet tehlikelerinin CBS kullanılarak yorumlanması: Balıkesir örneği. Balıkesir Üniversitesi Fen Bilimleri Enstitüsü Dergisi.

[B9-behavsci-16-01435] Faul F., Erdfelder E., Lang A. G., Buchner A. (2007). G*Power 3: A flexible statistical power analysis program for the social, behavioral, and biomedical sciences. Behavior Research Methods.

[B10-behavsci-16-01435] Feng J., Zhang C., Fang S., Zhao R., Wang H., Li D. (2025). Influencing factors of nurses’ disaster preparedness: A systematic review and meta-analysis. BMC Public Health.

[B11-behavsci-16-01435] Gençay G., Karadere K. (2024). Orman yangınları ve orman yakma suçunda toplumsal algının değerlendirilmesi (Balıkesir İli Örneği). Anadolu Orman Araştırmaları Dergisi.

[B12-behavsci-16-01435] Gündoğdu E., Özden S., Bekler T. (2020). Sındırgı fayi ve düvertepe fay zonu yakin civarinin kinematik ve sismotektonik özellikleri: Batı anadolu (Türkiye). Çanakkale Onsekiz Mart Üniversitesi Fen Bilimleri Enstitüsü Dergisi.

[B13-behavsci-16-01435] Hong Y., Chu H., Xie Z., Dalisay F. (2025). Before Helene’s Landfall: Analysis of disaster risk perceptions and preparedness assessment in the Southeastern United States in 2023. International Journal of Environmental Research and Public Health.

[B14-behavsci-16-01435] Huang W., Li L., Zhuo Y., Zhang J. (2022). Analysis of resilience, coping style, anxiety, and depression among rescue nurses on EMTs during the disaster preparedness stage in sichuan, china: A descriptive cross-sectional survey. Disaster Medicine and Public Health Preparedness.

[B15-behavsci-16-01435] Karatepe O. M., Avci T. (2017). The effects of psychological capital and work engagement on nurses’ lateness attitude and turnover intentions. Journal of Management Development.

[B16-behavsci-16-01435] Koyama T., Yamaguchi T., Matsunari Y. (2024). Public health nurses’ perceptions of their roles and activities throughout the phases of the fukushima nuclear disaster: A qualitative study. Nursing Reports.

[B17-behavsci-16-01435] Luo Y., Yan H., Tang Y., Wang S., Yang Z., Zhang T., Liu Y. (2025). Levels of nurse disaster preparedness: A systematic review and meta-analysis. Nurse Education in Practice.

[B18-behavsci-16-01435] Luthans F., Youssef C. M. (2004). Human, social, and now positive psychological capital management: Investing in people for competitive advantage. Organizational Dynamics.

[B19-behavsci-16-01435] Nikitara M., Kalu A., Latzourakis E., Constantinou C. S., Velonaki V. S. (2025). Training Nurses for Disasters: A Systematic Review on Self-Efficacy and Preparedness. Healthcare.

[B20-behavsci-16-01435] Özbey Ö., Bayraktar B. (2023). Balıkesir’in ekonomik yapısı. Balıkesir Üniversitesi İktisadi ve İdari Bilimler Fakültesi Dergisi.

[B21-behavsci-16-01435] Özcan F. (2013). Disaster preparedness and perceptions of nurses *(Publication No. 340806)*. Master’s thesis.

[B22-behavsci-16-01435] Özdemir S., Üzümcü L. Y., Altuntaş A., Rezaei S., Ünal C. S. (2025). The relationship between nursing students’ disaster response self-efficacy and psychological preparedness for disaster threat: A cross-sectional study. Sağlık Akademisyenleri Dergisi.

[B23-behavsci-16-01435] Pires E. G., Nogueira P., Henriques M. A., Arriaga M., Costa A. S. (2025). Environment disaster: A cross-sectional study of the determinants for the preparation of azorean nurses. Healthcare.

[B24-behavsci-16-01435] Said N. B., Chiang V. C. (2020). The knowledge, skill competencies, and psychological preparedness of nurses for disasters: A systematic review. International Emergency Nursing.

[B25-behavsci-16-01435] Said N. B., Molassiotis A., Chiang V. C. (2020). Psychological preparedness for disasters among nurses with disaster field experience: An international online survey. International Journal of Disaster Risk Reduction.

[B26-behavsci-16-01435] Said N. B., Molassiotis A., Chiang V. C. (2022). Psychological first aid training in disaster preparedness for nurses working with emergencies and traumas. International Nursing Review.

[B27-behavsci-16-01435] Silei G. (2014). Technological hazards, disasters and accidents. The basic environmental history.

[B28-behavsci-16-01435] Sohn H. G. (2015). Disaster classification for optimal disaster response in Korea. Journal of the Korean Society of Hazard Mitigation.

[B29-behavsci-16-01435] Şermet Kaya Ş., Erdoğan E. G. (2025). Disaster management competence, disaster preparedness belief, and disaster preparedness relationship: Nurses after the 2023 Turkey earthquake. International Nursing Review.

[B30-behavsci-16-01435] Türkdoğan Görgün C., Koçak Şen İ., McLennan J. (2023). The validity and reliability of the Turkish version of the psychological preparedness for disaster threat scale. Natural Hazards.

[B31-behavsci-16-01435] Ulupınar F., Altınel B., Aslan M. (2024). Investigating factors ınfluencing disaster preparedness perception of nurses in turkey: A meta-analytic approach. Disaster Medicine and Public Health Preparedness.

[B32-behavsci-16-01435] von Elm E., Altman D. G., Egger M., Pocock S. J., Gøtzsche P. C., Vandenbroucke J. P. (2007). The Strengthening the Reporting of Observational Studies in Epidemiology (STROBE) statement: Guidelines for reporting observational studies. The Lancet.

[B33-behavsci-16-01435] Wang Y., Liu Y., Yu M., Wang H., Peng C., Zhang P., Nian X., Jia Q., Li C. (2023). Disaster preparedness among nurses in China: A cross-sectional study. Journal of Nursing Research.

[B34-behavsci-16-01435] Zulch H. (2019). Psychological preparedness for natural hazards: Improving disaster preparedness policy and practice.

